# Characteristics of Arabic Websites with information on autism

**DOI:** 10.17712/nsj.2017.2.20160574

**Published:** 2017-04

**Authors:** Fahad M. Alnemary, Faisal M. Alnemary, Abdulrahman S. Alamri, Yassar A. Alamri

**Affiliations:** *From the Department of Human Development and Psychology/Special Education (Alnemary Fahad, Alnemary Faisal), University of California, Los Angeles, California, and from Department of Learning Technologies (Alamri), Universty of North Texas, Denton, Texas, United States of America*

## Abstract

**Objectives:**

To explore the characteristics of Arabic websites with information on autism spectrum disorder (ASD).

**Methods:**

The word autism in Arabic was entered into 2 popular search engines in September 2013 to locate the top 80 websites featuring the term. Websites were sorted using 10 characteristics, previously used to evaluate the characteristics of English websites with information on ASD.

**Results:**

Most websites were registered using a.com top-level domain (69%), were an individual’s site, forum, or blog (44%), and were updated after September 2012 (60%); they contained images or texts that seemed to persuade viewers to purchase products (43%); they provided information with the name of author(s) (64%); they described the basic characteristics of ASD; and they promoted various types of treatments, most of which lack empirical support (63%). However, few websites contained information with references to peer review resources (3%) or a warning statement that such information should not replace the opinion of a qualified professional (8%).

**Conclusion:**

Internet users may not find Arabic websites to be reliable sources to obtain information on ASD. Given the increased use of the internet, creation of websites that contain trusted information on ASD could potentially aid parents in accessing available services, help them learn about empirically validated interventions, and enable them to advocate for their children’s rights.

Improvement in awareness and diagnosis has led to an increase in the amount of information available about autism spectrum disorder (ASD).^1^ The internet contains much of this information^2^ and has become an important source for many parents of children with ASD.^3^,^4^ An examination of websites with information on ASD is essential, as they could potentially influence parents’ attitudes and values towards understanding and addressing their children’s needs.^5^ While English websites containing ASD information have been examined,^6^ there is no systematic information about websites in other languages, especially Arabic.

The Arabic language is the mother tongue of over 300 million people across 22 countries in the Middle East and North Africa. These countries include Algeria, Bahrain, the Comoros Islands, Djibouti, Egypt, Iraq, Jordan, Kuwait, Lebanon, Libya, Morocco, Mauritania, Oman, Palestine, Qatar, Saudi Arabia, Somalia, Sudan, Syria, Tunisia, the United Arab Emirates, and Yemen.

Awareness of ASD is relatively new in the Arab world, yet with increasingly easier accessibility to the internet, personnel interested in ASD (both healthcare providers and families of affected individuals alike) appear to be willing to use online resources and ‘look up’ information. A brief examination of the search trends in a major online search engine shows an increase in searches about ASD over time, as well as peaks of entries for ‘autism’ and ‘ASD’ corresponding with major local newspaper headlines. In addition, recent investigation has revealed that many Arabic-speaking parents of children with ASD find the internet as a major source, and in most cases the only source to learn about ASD and available treatments and services.^7^

Given the increased use of the internet in the Arab world to search for information about ASD, examination of such websites is warranted. In the present study, we attempt to bridge the gap in the literature by examining characteristics of Arabic websites with information on ASD. Our research question was: what are the characteristics of Arabic websites with information on ASD.

## Methods

### Research strategy

In September 2013, the word autism in Arabic (دحوتلا) was used to locate the top 80 websites returned by 2 search engines: Google (http://www.google.com) and Yahoo (http://www.yahoo.com). In addition, the search engines were searched in 2 Arab countries (Egypt and Saudi Arabia) to uncover any local or regional search results that would have otherwise been missed from the initial search. To control for a search history effect, we also deleted the search history in the computer and then conducted the search using a newly loaded web browser (namely (i.e.), Firefox). Overlaps across the 2 search engines were observed and resolved by eliminating repeated websites, resulting in final sample of 118 websites.^8^

### Data extraction

Websites were sorted using 10 variables, previously used to evaluate the characteristics of English websites with information on ASD.^6^,^8^ These characteristics included nature, top-level domains, attribution, currency, advertisement, disclaimer, authorship, feedback, general information, and promotion of intervention.

The nature of the website was coded into one of the following categories: governmental if the website was associated with a governmental agency; personal if the website contained information based on an individual’s or a group of users’ opinion; online informational if the website included information about different health-related topics; news if the website was sponsored by a national or local television or newspaper; university if the website was sponsored by a college or university; nonprofit organization if the website was hosted by a nonprofit organization dedicated to furthering a particular social issue or advocating for a particular point of view; and service provider if the website was sponsored by a clinic or center or school for people with ASD. All other websites were coded as others.

Top-level domain was coded into one of 6 categories:.com,.org,.edu,.net,.gov, and other. Websites with attribution provided information referenced to peer-reviewed materials. A website was coded current if there was evidence it had been updated within the previous year (i.e., after September 2012). A website with advertisement contained an image or text persuading viewers to purchase certain products. A website containing authorship provided information with the name of author(s). A website with a disclaimer displayed a general statement limiting liability. A website with feedback contained a method of contacting an individual associated with that website. A website with general information described the basic characteristics of ASD.

A website with promotion of intervention contained information about intervention that might be used with individuals with ASD. Interventions were coded into the 5 categories based on the autism healthcare research and quality report. These categories included Behavioral (for example (e.g.), Applied Behavior Analysis, The Early Start Denver Model), Educational (Treatment and Education of Autistic and Related Communication Handicapped Children), Medical and Related interventions (e.g., Hyperbaric oxygen therapy, diets, vitamins), Allied health (e.g., speech therapy, occupational therapy), and complementary and alternative medicine (massage, and acupuncture). The first 2 categories had more evidence to support their use as compared to the other categories, which had no (medical and related interventions and complementary and alternative medicine interventions) to little support (allied health).^9^

## Results

**Table 1** presents the characteristics of Arabic websites. Most websites were an individual’s site, forum, or blog (44%), were registered using a.com top-level domain (69%), had ben updated after September 2012 (60%), listed methods of contacting an individual associated with the website (82%), contained images or texts that seemed to persuade viewers to purchase products (43%), provided information with the name of the author (64%), described the basic characteristics of ASD (63%), and promoted various types of treatments as effective (66%). Promoted treatments included behavioral interventions (42%), educational resources (18%), medical and related interventions (43%), allied health interventions (34.8%), and complementary and alternative medicine therapies (9%). Few websites attributed their information to peer reviewed resources (3%) and included warning statement that such information should not replace the opinion of a qualified professional (8%).

**Table 1:** Characteristics of Arabic websites with information on ASD (N=118).

Website characteristics	(%)
Country of website’s origin (known)	(68.0)
*Nature*	
Organizations	(3.0)
Individuals sites	(44.0)
Health information websites	(7.0)
Governments	(1.0)
Service providers	(4.0)
University	(2.0)
News	(33.0)
Other	(6.0)
*Top-level domain*	
.com	(69.0)
.org	(9.0)
.net	(11.0)
.edu	(2.0)
other	(9.0)
Attributions	(3.0)
Authorship	(64.0)
Currency	(60.0)
Advertisement	(43.0)
Disclaimer	(8.0)
Feedback	(82.0)
General info	(66.0)
*Treatment*	(63.0)
Behavioral	(42.0)
Educational	(18.0)
Allied health	(40.0)
Medical and related interventions	(43.0)
Complementary and alternative medicine	(9.0)

## Discussion

The current study examined Arabic websites with information on ASD. Overall, websites primarily featured personal content and information with little to no references to peer reviewed resources or warning statements that such information should not replace the opinion of a qualified professional. These findings are somewhat consistent with existing research from Western countries indicating that ASD information on English websites was difficult to access and lacked empirical support and that websites with a.gov top-level domain were associated with higher quality information.^6^ In addition, some of the websites promoted the use of non-medical and biomedical interventions simultaneously, which is in line with evidence supporting their effectiveness when used concurrently.^10^ However, other websites recommended the use of only one ASD intervention that had little to no support, including cultural and religious treatments (e.g., visiting a religious healer, goat milk, honey) or only medical and related interventions (e.g.., chelation therapy, hyperbaric oxygen therapy). This could potentially lead to the acquisition of erroneous information and, even worse, the implementation of therapies that are not evidence-based or that might even be harmful. Our finding suggests that consumers should not use Arabic websites as a reliable source to obtain information on ASD.

Findings from the present study underscore the importance of creating websites for users searching the internet for Arabic information on ASD. Such websites may describe the wide-ranging features of individuals with ASD, the processes of obtaining an ASD diagnosis and accessing local services, treatments with empirical support, and the legal rights of individuals with ASD and their families. Then parents would have information to potentially advocate for their children’s rights and access available services as well as refrain from utilizing controversial and unsupported treatments, resulting in savings in time, energy, and money.

The results of this study should be interpreted in the context of the following limitations. First, the sample might not be representative of all websites containing Arabic information on ASD. Using different search strategies (e.g., multiple terms in additional search engines) might have yielded more robust results. In addition, the reported search date is September 2013, nearing 3 years ago. One may argue, given the speed with which new websites can be created and the changes within the ASD community (especially changes to diagnostic criteria in The Diagnostic and Statistical Manual of Mental Disorders), search results are likely to have changed since the search was first conducted. However, a brief and recent search of Arabic websites containing information on ASD yielded findings consistent with those of this study. The search included websites of major governmental agencies (e.g., the Ministry of Health and the Ministry of Education). These agencies are responsible for the healthcare and welfare of individuals with ASD in the Arab World countries. Despite these limitations, results from this study offer important insights into Arabic websites containing information on ASD.

In conclusion, internet users should not rely on Arabic websites to obtain information on ASD. Given that the internet is a major source to learn about ASD, creating websites that contain best practices information on ASD is an important step to make trusted information available for parents, potentially supporting them in accessing available services and advocating for their children’s rights.
